# Science identity development: an interactionist approach

**DOI:** 10.1186/s40594-018-0149-9

**Published:** 2018-11-30

**Authors:** Ann Y. Kim, Gale M. Sinatra

**Affiliations:** 1 Department of Human Development, California State University, Long Beach, USA; 2 Rossier School of Education, University of Southern California, Los Angeles, USA

**Keywords:** Identity development, STEM identity, STEM learning

## Abstract

In this introduction for this Special Issue we discuss the need for the investigation of science identity with an emphasis on the environment. As such, we propose taking an interactionist approach; one that examines the person in interaction within their environment (Adams & Marshall, 1996). The Special Issue highlights the role of psychology constructs, such as interest and belonging that are deeply relevant and ultimately inform students’ science identity development. The Special Issue includes six articles: this introduction, four empirical papers investigating the psychological experiences of students in various science spaces with a focus on the interactions between the individual and the context, and a commentary. Each contribution emphasized how the context either afforded or did not afford that sense of belonging to develop in students. The collection of articles were inspired by a symposium on the topic of STEM identity development that was presented at the 2017 annual meeting of the American Educational Research Association (AERA) in San Antonio, TX.

## Science identity development: an interactionist approach

The question of how to encourage all students to pursue and actively engage in science has been at the foreground of interest in STEM education for some time. Some scholars have argued that sociodemographic differences among students are the primary explanation for differences in science achievement and ultimately, science pursuit (e.g., Morgan et al. 2016). From this vantage point, the focus of investigation has been on how to support individuals to adapt to the science context. This work is important and has made significant contributions to our understanding of belonging in the STEM disciplines (for a review of sense of belonging in computer science, see Cheryan et al. 2015). However, this perspective places the onus on the student to develop the pre-requisite knowledge, skills, interests, and abilities required to be successful in science majors and careers. In this Special Issue, *Science Identity Development*: *An Interactionist Approach*, we argue that the time has come for a shift in focus in thinking about science identity. STEM education researchers need to explore ways in which science identity is fostered or thwarted by the context in which students experience science. Therefore, the articles in this Special Issue provide a closer examination of the interaction between the individual student and the context that affords (or restricts) entry into and persistence in science.

Recent investigations have proposed student identity as a means of understanding science pursuit differences. Scholars have addressed this topic of identity from many approaches. Some propose frameworks for understanding the psychological and developmental process of identity development in education more generally (see Kaplan and Flum 2012; Dynamic Systems Model of Role Identity, Kaplan and Garner 2017), while others have sought to understand domain-specific identities, such as a science identity (Carlone and Johnson 2007). Despite the variation in approach, much of this research tends to focus on how the individual fits into the science environment. Studies focus on how the individual feels s/he belongs in the science environment without sufficient examination of how the culture and context of the science environment is welcoming (or not welcoming) the individual into the field, particularly those individuals who have not historically been included in science fields in representative numbers.

The purpose of this Special Issue is to bring together a set of articles that explore the environment and individuals’ psychological experiences in interaction within science environments. These interactions inform and shape the development of science identities. The contributing articles examine implicit or explicit signals of exclusion or welcoming afforded to the student from the science culture and context. These signals are seen through the lens of the individual’s perspective to shed light on how the environment encourages or discourages the development of science identity. The empirical papers included in this Special Issue have investigated this topic in a range of psychological constructs, developmental levels, and educational contexts from elementary schools to undergraduate education. Collectively, they demonstrate how important it is to explore identity development at every level of education. By examining students’ experiences in interaction with their STEM contexts, these articles provide new insights and perspectives for advancing our understanding of science identity development.

## Why science identity?

An identity is a “general sense of self with reference to groups or particular content” (Renninger 2009, p. 109). In other words, an identity develops in relation to and through interactions with others, making identity inherently social in nature. Accordingly, the experiences in science educational settings, such as experiences with peers, teachers, professors, mentors, and materials in those settings, inform individuals’ understandings of self. We choose to focus on science engagement and its relation to science identity development because much research has emphasized that not all students feel welcome in STEM disciplines.

In the past decade, science education scholars have been examining science identity with the framework proposed by Carlone and Johnson (2007). The framework suggests student science identity is made up of three dimensions: competence, performance, and recognition. Competence refers to having scientific knowledge as well as motivation to “understand the world scientifically” (p. 1190). Performance refers to being able to demonstrate the scientific knowledge to others. Recognition refers to both self-recognizing as well as meaningful others recognizing one as a “science person.” According to this model, a science identity is composed of different combinations of these dimensions. For instance, a student may be able to perform activities that scientists do, show competence in that performance, but may not be recognized as a “science person” by others. Whereas, another student may be recognized as a “science person” who may in fact have lower competence and lower performance than the former individual.

Whereas the Carlone and Johnson framework is an important and useful framework, the authors referred to their framework as “still evolving” (p. 1207), and recognize gaps in the framework. One gap seems in the description of the dimension of recognition. Carlone and Johnson (2007) emphasize the importance of recognition from those they define as “meaningful, scientific others.” However, possibly because of the lack of explicitly recognizing science identity as a social identity, the potential for impact of others such as peers is not fully explored. This gap in recognizing others who impact science identity formation becomes more apparent when examining the literature on perceptions of who is a scientist. In a study with undergraduate students in a biology class, male students nominated their male peers as being more knowledgeable and female students nominated their male and female peers equally in a biology class, even after controlling for class performance and outspokenness (Grunspan et al. 2016). This suggests that even when the performance of two capable biology students is the same, a female and a male student are recognized differently and in turn will be treated differently. In another study with Amazon Mechanical Turk participants, women who looked feminine were less likely to be rated as scientists (Banchefsky et al. 2016). The photos used were of actual women scientists. These two research studies demonstrate that individuals hold perceptions that men are more likely and women are less likely to be scientists. These gender stereotypes likely impact young women pursing science.

Another limitation of the framework is that it does not place sufficient emphasis on the role of the environment when investigating science identity development. Recognition from others is important when a student is explicitly aware of the recognition or the lack thereof. However, environmental influences can be more subtle. Master et al. (2016) tested manipulating wall decorations (*Star Wars* posters versus art) and items displayed around computer science classrooms (science fiction books versus general magazines) and found these small differences were associated with computer science interest. Interestingly, female students and some male students tended to prefer the non-stereotypical classroom. This suggests that making the environment more welcoming extends beyond explicit recognition of individual students. It also suggests that there are opportunities to support the development of a science identity in a wider range of students through environmental cues. When students feel included, they are more likely to continue engagement in science (e.g., Master et al. 2016; Wilson et al. 2015).

A new framework proposed by Kim et al. (2018) examined science identity of young women through a social identity framework. Kim and colleagues applied social identity theory (Tajfel and Turner 1979) to review the experiences of middle and high school students regarding STEM. Young women were still experiencing parents, teachers, and peers who communicated that the young women did not belong in STEM classes and environments, despite their high achievement and interest. On a more optimistic note, all the efforts to change the environment met with positive results, with programs intended to encourage interest and engagement in STEM reporting positive changes or no change because the young women were already highly interested and engaged. After reviewing the literature with attention on the role of the environment, Kim and colleagues argue the gender differences in STEM pursuits are less related to young women's lack of interests or achievement. Rather, many of them are made to feel unwelcome, that STEM is not a place for women, or that those who pursue STEM are men. These messages indicated that there were clear perceptions of who is in the STEM-ingroup and who is in the STEM-outgroup. These are messages that students receive, internalize, and may act upon in terms of future pursuits.

Kim et al. (2018) suggest that these ingroup-outgroup boundaries in science must be shifted. Some of those boundaries are created by societal perceptions regarding the nature of science and scientists. Research shows that it is not just adults who hold stereotypical views of what a scientist looks like. In a study by Archer et al. (2010), 10-year-old students in London schools described various perceptions about science and scientists. These young students shared detailed beliefs not only about what kind of person engaged in science (“bald, wears goggles, etc.” p. 633) but also their beliefs were seemingly irrelevant to science, such as that science and fashion did not mix (expressed in an all-boys interview group). These images contribute to setting a boundary of who belongs in science and who does not. Social comparison is a pastime of adolescent students, who are beginning to seriously reflect on their personal and occupational goals (Arnett 2015). Students compare themselves to their classmates, and those who do not “measure up” by whatever standards they deem are likely to move away from the ingroup. If the ingroup is scientists and they are considered “geeky” or “nerdy,” that could create a barrier for a student who wants to be scientist but does not want to be labeled as a nerd.

As students learn about the ingroup-outgroup boundaries from the environment, the perceptions of these boundaries can be changed through enacting changes in that environment. In a 1-week program for elementary students who made 3D dioramas of female scientists they were studying, the researchers reported parents of the boys asked if their sons could make dioramas of male scientists (Teske et al. 2014). This suggested the parents held the assumption that a program engaging students in learning about female scientists was only meant for girls. The parents were informed that their sons were asked to make dioramas of female scientists because the program intended to convey the important contributions of women scientists, as it is often overlooked. Before the end of the week, the boys were excited and boasting to one another about the accomplishments of the female scientists they had selected to represent in their dioramas. This demonstrates the importance of the messages children receive from the parents, teachers, peers, and objects in their environment regarding what kind of person may be a scientist.

## This special issue

Given the importance of considering context or environment in the development of science identity, we sought out scholars who were focused on these issues in their research. In the process of seeking out scholars investigating science identity development, we organized a symposium on the topic at the 2017 annual meeting of the American Educational Research Association (AERA) in San Antonio, TX. After a successful symposium, most of the scholars agreed to contribute to this Special Issue. We recognize that there are many other scholars who are making important contribution in this area, whom we did not include. It should be noted that this Special Issue offers only a sampling of the work that is ongoing in this field. We hope that the publication of this Special Issue will encourage those already pursuing this work as well as new scholars to pursue this topic. There is so much important work to be done from a variety of theoretical perspectives and research traditions.

As the focus of this Special Issue is the development of science identity within an environment, we are taking an interactionist approach. An interactionist approach examines the person in interaction within their environment (Adams and Marshall 1996). The Special Issue highlights the role of psychology constructs, such as interest and belonging that are deeply relevant and ultimately inform students’ science identity development. But, importantly, each contribution emphasizes how the context either afforded or did not afford that sense of belonging to develop in students. The Special Issue includes six articles: this introduction, four empirical papers investigating the psychological experiences of students in various science spaces with a focus on the interactions between the individual and the context, and a commentary.

The empirical articles begin with work by Vongkulluksn et al. (2018) in an elementary school setting. They report findings from observing 3rd- to 6th-grade students’ engagement in a *design*-*based makerspace* over the course of a school year. Drawing on social cognitive and interest development perspectives, Vongkulluksn and colleagues investigated the change in students’ self-efficacy, emotions, and situational interest in this unique design-based learning environment. Students were required to address a real-world problem by designing a solution. Their study demonstrated how students situated in this new environment struggled to deal with frustrations and loss of interest, hence competency was challenged and successful performance took on a different form (e.g., how one reacted to failure). Makerspaces are at times discussed as magical spaces that afford anyone interested in science positive experiences. However, Vongkulluksn and colleagues’ found that self-efficacy is important for interest to flourish. They highlight that students’ ambitions (to build a robotic prosthetic arm) may have outpaced their abilities, time, and resources, and put even the most typically successful of students on their heels. They emphasize the need to support students’ creativity and independence while at same time helping students craft realistic projects that do not discourage them from future STEM engagement.

Moving developmentally to a secondary school setting, Vincent-Ruz and Schunn (2018) critically examine the internal perceptions and external reinforcement of a science identity among middle school and early high school students. Conceptually, Vincent-Ruz and Schunn directly question Carlone and Johnson’s (2007) model that competency beliefs are a dimension of science identity. Using survey data from 6th, 7th, and 9th grade students from 19 public schools, Vincent-Ruz and Schunn examine whether science identity is different from other attitudinal constructs commonly investigated as components of identity, specifically, fascination, valuing science, and competency beliefs. By investigating whether science identity is predictive of student choices to engage in science experiences, they suggest the interactional nature of environment and science identity. In other words, science identity will impact the science environment one chooses, and then the environment reciprocally impacts identity development.

Next, we turn to the higher education context. Robnett et al. (2018) examine the value of mentoring relationships for the development of undergraduate students’ scientist identities. Mentors are crucial in the pursuit of research. They are key figures in the environment for novice scientists and are significant meaningful others that will provide the recognition needed for pursuing a science career. Robnett and colleagues identify three different kinds of mentoring: instrumental, socioemotional, and negative mentoring. Using data collected from 66 dyads of undergraduate mentees and their faculty mentors, Robnett and colleagues examined how perceptions regarding the quality of the mentor-mentee relationship relate to the mentee’s science identity.

Continuing with a higher education context, Seyranian et al. (2018) examine how *belonging uncertainty*, that is, doubts about whether you belong in a field, is exacerbated for women who are in small numbers in fields like physics. Seyranian and colleagues collected survey data at the beginning and at the end of the semester from three sections of an undergraduate physics course. Interestingly, the women in their study reported they felt a stronger sense of belonging at the university than they did in physics, showing the strong impact of environment on sense of belonging. They also showed that identification with the field of physics is related to psychological well-being (*flourishing*) and academic achievement. Women who did identify with physics tended to not only perform well, but “flourish,” which is a construct from social identity theory (Tajfel and Turner 1979) that refers to an optimal range of human functioning that is associated with “generativity, growth, and resilience” (Fredrickson and Losada 2005, p. 678).

The issue concludes with commentary from Martin-Hansen, a science education scholar. Martin-Hansen reviews the articles in this special issue and discusses how both internal and external factors play roles in the development of STEM identities in educational settings. Martin-Hansen concludes with suggestions for how professional development and hiring practices can change in order to further recruit and keep diverse persons in STEM careers.

We find three overarching themes of the Special Issue. Firstly, we maintain that *all individuals have the potential to develop a science identity*. Secondly, *science identity is a developmental process that unfolds over time*. Lastly, *the environment informs the identity the individual develops*.

With regards to the first theme, work from all four contributors in this issue demonstrate that students of different age groups, genders, races, ethnicities, and socioeconomic statuses all are in the process of developing some form of a science identity. It is important to keep in mind that not all students want to become or should become STEM majors or professionals. The world needs artists, poets, entrepreneurs, and a host of other professionals. Our point is identification with science can include identification with thinking and reasoning like a scientist, skills that are useful in a variety of professions and in day to day life. The student in Vonkulluksn et al.’s study who wanted to develop a robotic arm could become a scientist or an engineer, but also an architecture who designs handicapped accessible housing. The point is that a science identity can imply seeing oneself as someone who engages in thinking, reasoning, and problem solving systematically to find creative solutions to a problem.

Secondly, a science identity develops over time. Vongkulluksn and colleagues report research from elementary students and their early experiences in the makerspaces. These experiences will likely inform how the students approach their future science engagement. Vincent-Ruz and Schunn found that student science identity was a predictor of out-of-school science experiences. Just as personal and psychological identity develops over time, science identity unfolds as individuals who engage in science are afforded (or denied) positive experiences and opportunities. The articles in this Special Issue demonstrate how individuals carry past experiences forward which informs not only their current identity but also their future identities. Sensitivity to the developmental nature of science identity will help science educators support that development at all ages and educational levels.

Lastly, the environment has an important interactional impact on science identity development. Students experienced a new environment in Vongkulluksn et al.’s study in the form of a makerspace, which initially was not without its challenges. Mentors, teaching, and university instructors are also elements of the environment that help shape students’ science identity development. Robnett and colleagues found students to have higher levels of science identity when they considered mentors engaging in more instrumental and socioemotional mentoring. Seyranian et al. showed the reciprocal nature of the interactionist perspective in that students who had greater content knowledge of physics felt higher levels of belonging, but only those who felt welcome were among those who stayed long enough to accrue such knowledge. Taken together, these studies shine a light on how the environmental is a powerful force shaping science identity development. Fortunately, every element in a science environment from the materials that are present to the choice of activities, to how mentors, teachers, and peers interact with students can all be adjusted to make the environment more welcoming and supportive.

## Interactionist framework

We invite the reader to critically consider the role of the environment in interaction with students’ psychological experiences as contributors to science identity development. Given the work included in this Special Issue as well as previous literature on science persistence, we encourage the exploration of *an interactionist approach to science identity development* that emphasizes both the individual and the science context for considering how science identity may develop. We posit that all students develop a science identity that is either *approaching* or *avoiding* science as they have different science experiences, either explicitly or implicitly. Some experiences are encouraging and reinforcing of a science-approach identity, such as programs intended to develop interest and positive attitudes toward science. These are afforded by experiencing success in a makerspace or having a positive mentoring experience. These experiences encourage students’ to approach science engagement and positive science identity development. Other experiences may encourage a science-avoid identity in that they discourage students from engaging in science, such as experiencing academic sexism, or poor fit with mentors, or feelings of frustration and low self-efficacy in a makerspace learning environment. As environments are constructed to be more supportive of all types of students, perhaps more individuals would approach rather than avoid science, broadening the sciences and our conceptions of science identity.

## Conclusion

With only a few articles to share in this Special Issue, we acknowledge that there are many aspects of science identity research that are left unaddressed. One significant body of work missing is that examining science identity development using intersectional approaches. Intersectionality recognizes characteristics such as race-ethnicity, gender, low socioeconomic status, sexual orientation, as separate disadvantaging factors (Delgado and Stefancic 2017). Given our emphasis on considering the impact of the environment, it would be remiss of us not to acknowledge the need to examine the different experiences of students who hold intersectional identities in science spaces.

Missing from this issue are works that investigate teaching and learning science, including those that focus on the development of science content knowledge and the consideration of content knowledge being a key factor in the development of a science identity. We have not included any of those studies as we are attempting to propose an alternative way of thinking about the development of science identity that emphasizes the role of the environment and that examines attitudes regarding science-related experiences. We encourage conceptions of science identity that move beyond identity as something that one has or does not have. Instead, as we discussed in our interactionist framework, we propose that all students should be recognized as having the potential to develop a science identity. Similar to how educators encourage students not to think of themselves as “a math person” or “not a math person,” all students should be encouraged to approach science (rather than avoid it). We hope that readers of this Special Issue will continue to expand the field’s understanding of how science identity develops and also how parents, teachers, peers, and materials play key roles in support of identity development. Lastly, we encourage researchers to consider new ways to measure science identity development that reflect an interactionist point of view.

In conclusion, we hope that readers will learn from the contributions of this Special Issue the importance of considering how experiences in science environments function as sources of information for students when they are determining their place as a scientist, so that the concept of science identity in the future becomes irrelevant, as anyone who wants to engage in science is given the opportunity to do so and is welcomed to the endeavor.
